# Uptake of environmental DNA in *Bacillus subtilis* occurs all over the cell surface through a dynamic pilus structure

**DOI:** 10.1371/journal.pgen.1010696

**Published:** 2023-10-10

**Authors:** Alexandra Kilb, Marie Burghard-Schrod, Sven Holtrup, Peter L. Graumann

**Affiliations:** Fachbereich Chemie und Zentrum für Synthetische Mikrobiologie, SYNMIKRO, Philipps-Universität Marburg, Marburg, Germany; The University of Texas Health Science Center at Houston, UNITED STATES

## Abstract

At the transition to stationary phase, a subpopulation of *Bacillus subtilis* cells can enter the developmental state of competence, where DNA is taken up through the cell envelope, and is processed to single stranded DNA, which is incorporated into the genome if sufficient homology between sequences exists. We show here that the initial step of transport across the cell wall occurs via a true pilus structure, with an average length of about 500 nm, which assembles at various places on the cell surface. Once assembled, the pilus remains at one position and can be retracted in a time frame of seconds. The major pilin, ComGC, was studied at a single molecule level in live cells. ComGC was found in two distinct populations, one that would correspond to ComGC freely diffusing throughout the cell membrane, and one that is relatively stationary, likely reflecting pilus-incorporated molecules. The ratio of 65% diffusing and 35% stationary ComGC molecules changed towards more stationary molecules upon addition of external DNA, while the number of pili in the population did not strongly increase. These findings suggest that the pilus assembles stochastically, but engages more pilin monomers from the membrane fraction in the presence of transport substrate. Our data support a model in which transport of environmental DNA occurs through the entire cell surface by a dynamic pilus, mediating efficient uptake through the cell wall into the periplasm, where DNA diffuses to a cell pole containing the localized transport machinery mediating passage into the cytosol.

## Author summary

Horizontal gene transfer (HGT) is important for bacterial evolution and the adaption to new environments. Through HGT, genes can be transferred between bacteria, which can lead to antibiotic resistance development, especially critical with regards to pathogenic bacterial species. One mechanism of HGT is natural competence, through which bacteria can take up DNA under natural conditions and incorporate it into their chromosomes. For DNA uptake, a complex will be formed that is conserved among Gram-positive and Gram-negative bacteria, in spite of different envelope architectures. We show that in *Bacillus subtilis*, where the part of the uptake complex spanning the cell membrane localizes to a single cell pole, transport through the cell wall is mediated by a visible pilus structure that can extend and retract to pull DNA into the periplasm. Thus, DNA uptake from the environment is not limited to polar sites, but is channelled into the cytosol at the pole. Single molecule dynamics of ComGC revealed a static, likely pilus-bound, and a mobile fraction of ComGC within the cell membrane. Addition of environmental DNA increased the static fraction of molecules, showing that pilus dynamics respond to DNA binding.

## Introduction

Prokaryotes are known to colonize almost every place on planet earth, including environments with extreme environmental conditions that require specific traits to survive [1]. Bacteria present the largest domain of life, and are known for their adaptability to changing environmental conditions. There are two main drivers for genomic adaptation; one is horizontal gene transfer (HGT), the other accumulation of beneficial mutations based on random base changes in DNA, within large population sizes. HGT is facilitated by the uptake of foreign DNA, by conjugation (direct transfer involving a pilus structure), transduction (via phage infection) or competence [2]. Naturally competent bacteria can take up environmental DNA through the cell envelope, and become transformed when DNA is incorporated into their genome, which is a physiological and genetical trait of many bacterial strains [3]. Alternatively to chromosome integration, taken up DNA can be recombined to yield plasmid DNA, in case such DNA has been available [4], or can be metabolized in the cell as a source of phosphate, nitrogen and carbon [2, 5, 6]. Some bacterial strains, including pathogens, are naturally competent, and can thus obtain antibiotic resistance genes through natural transformation leading to drug resistance; therefore, competence is of clinical relevance [7, 8]. In 2014 it was reported that 80 bacterial strains were identified to be naturally competent under various conditions, but likely, there are many more species that can become competent under conditions that are not yet known [9]. The Gram-negative bacterium *Vibrio cholerae* takes up exogenous DNA on chitin surfaces [10, 11], while *Neisseria gonorrhoea* shows constitutive expression of competence [12]. In other bacteria, like in the Gram-positive bacterium *B*. *subtilis* or the Gram-negative bacterium *Haemophilus influenzae*, the development of competence is induced by e.g. conditions that limit growth (reviewed in [3, 13]). This variability of competence-induction has the purpose to serve the organisms in their special niches [3]. In some organisms, like the human pathogens *N*. *gonorrhoeae* and *H*. *influenzae*, preferences for specific DNA uptake sequences were identified, which in this case consist of 10 base pairs [14]. In *B*. *subtilis*, sequence preference for uptake is not known. During the state of competence around 100 genes are upregulated in this organism, of which 20 are essential for DNA uptake [15, 16]. Environmental DNA is taken up by a multiprotein complex, which differs between bacterial strains, but is similar in its basic structure. In Gram-negative bacteria, DNA has to overcome an additional outer membrane, while in Gram-positive bacteria DNA has to pass a thick 30–50 nm peptidoglycan layer [17]. The proteins forming the uptake complex in *B*. *subtilis* were identified in a systematic transposon screen [18]. It is assembled in just 2–20% of cells within a culture, dependent on the strain [19]. Thus, cell fates such as competence or sporulation are specialization strategies, which are pursued by a subpopulation of cells. Decisions are based on bistable switches, an intriguing phenomenon in bacterial populations [20]. The regulatory system of competence is induced via quorum sensing, i.e. by secreted peptide factors produced by the cells, leading to the stabilization of the master transcription factor ComK, whose gene, *comK*, is repressed by ROK (Repressor of competence). The deletion of *rok* results in up to 80% of cells reaching the state of competence [21]. The DNA uptake complex is composed of late competence proteins encoded by four different operons [22–25]. Intriguingly, parts of the machinery have been shown to be located at one, or rarely at both cell poles: ssDNA transporting membrane protein ComEC and putative ATPase ComFA are polarly located, as are many proteins that accept incoming DNA and mediate its incorporation into the chromosome via homologous recombination [4, 26–30]. These findings strongly suggest that DNA uptake–at least through the cell membrane—occurs at a specific site in competent cells. Accumulating evidence suggests that Com proteins localize specifically near the poles due to a diffusion and capture mechanism [29, 31], with a true “anchor” protein still being unknown. When cells are converted into round spheroplasts, single positions within the membrane were found to persist, revealing that the assembly is highly stable and does not require the cell pole for its maintenance [28]. Immunofluorescence microscopy revealed that, like other competence proteins, ComGC, the major pilin forming the basis of the pseudopilus structure, also localizes at poles and cell centre. Therefore, it was proposed that cells may contain just one active, assembled competence machinery and non-assembled single competence proteins [28].

Common to DNA uptake systems in Gram-negative bacteria is periplasmatic uptake occurring by retraction of a pilus structure [2]. The term periplasm for Gram-positive bacteria is used here as a description for the extracellular space between the cell wall and the cell membrane, which is thought to serve a similar function as the Gram-negative membrane-enclosed periplasm. For *B*. *subtilis*, the existence of a “pseudopilus” was shown, composed of a major pilin (ComGC) and some minor pilins (ComGD, ComGE, ComGG). These membrane proteins are processed in their N-terminal region to gain the ability of formation of the pilus structure, by ComC, the pre-pilin peptidase. An intramolecular disulphide bond is introduced into ComGC by an oxidoreductase pair BbbD und BdbC [32]. Loss of the cell wall relieves the essential nature of the ComG operon for DNA uptake [22], suggesting that the complex, whose nature is still unclear, is essential to move DNA through the cell wall. In other bacteria, pili have been labelled *in vivo* by introducing a cysteine substitution in the major pilin and subsequent labelling with maleimide dyes [33]. With this method, pilus structures in the human pathogens *V*. *cholerae* or *Streptococcus pneumoniae* were identified and stained. It was possible to follow the DNA uptake in real time and it was shown that pilus retraction is essential for DNA uptake in these bacteria [34, 35]. Contrary to Gram-negative systems, a retraction ATPase is missing within the systems of Gram-positive bacteria. It was therefore proposed that also ComGA, the assembly ATPase in *B subtilis*, might have a bifunctional role, or that cytosolic DNA helicase ComFA might induce retraction, facilitating the uptake of environmental DNA [2]. After uptake into the periplasm, double stranded DNA binds to the DNA receptor ComEA at its C-terminus [36, 37]. This domain is composed of Helix-Hairpin-Helix motives which interact with DNA without any sequence specificity. In recent work, tracking of fluorescently labelled DNA in *B*. *subtilis* showed that taken up DNA is localized in the periplasm before it enters to the cytosol [31, 38]; the periplasm thus acts as a reservoir for DNA like in *V*. *cholerae* [39]. The DNA might itself be mobile within the periplasm or might move together with ComEA. After degradation of one DNA strand, single-stranded DNA can enter to the cytosol through the channel protein ComEC, which is localized at the cell pole in competent cells, and may have nuclease activity *in vivo* for generating ssDNA [40].

A key missing question is the localization and architecture of the complex mediating cell wall passage of DNA in *B*. *subtilis*, which we show to have true pilus properties and to respond to interaction with environmental DNA.

## Methods

### Growth conditions

*Escherichia coli* and *Bacillus subtilis* cells were grown in lysogeny broth media (LB) for liquid cultures and on LB-Agar plates (1.5% agar) with respective antibiotics (5 μg/ml chloramphenicol [cm], 100 μg/ml spectinomycin [spec], 25 μg/ml lincomycin and 1 μg/ml erythromycin [MLS], 25 μg/ml kanamycin [kan]) and supplements (0.01 mM, 0.05 mM, 0.1 mM, or 0.5 mM Isopropyl-β-D-thiogalactopyranosid [IPTG]). For liquid cultures of *E*. *coli*, 100 μg/ml ampicillin [amp] was added to the media and the culture was incubated on a shaking platform with 200 rpm at 37°C. Liquid cultures of *B*. *subtilis* were grown at 30°C on a shaking platform (200 rpm) supplemented with the required antibiotics. To verify the successful integration at the *amyE*-locus in *B*. *subtilis*, LB-Agar plates were supplemented with 1% starch (w/v). The expression from the *amyE*-locus with a *hyperspanc* promoter was induced by IPTG. For competence induction in *B*. *subtilis*, the modified competence medium according to Spizizen was used [41]. A volume of 100 ml of 10x MC-media was composed of: 14.01 g K_2_HPO_4_ x 3 H_2_0, 5.24 g KH_2_PO_4_, 20 g Glucose, 10 ml trisodium citrate (300 mM), 1 ml ferric ammonium citrate (22 mg/ml), 1 g casein hydrolysate, 2 g potassium glutamate. Sterile filtered media was stored at -20°C. For inoculation of a 10 ml culture, 1 ml of 10x MC media, 0.333 ml of sterile 1 M MgCl_2,_ the respective antibiotics or supplements were added and filled up with autoclaved water. Cells were grown to competence on a shaking platform (200 rpm) at 37°C.

### Strain constructions

To investigate point mutations of ComGC, the gene was amplified from chromosomal DNA of PY79 with primers listed in S1 Table. The gene was cloned into pDR111 using *Nhe*I and *Sph*I sites, for an ectopic expression at the *amyE*-site (PLASMIDS SEE S2 Table). The plasmid does not contain a ribosomal binding site (RBS); therefore, 24 bp encoding a RBS were added N-terminally to all constructs by a second vector-PCR (sequence, including **RBS**: GATTAACTAATA**AGGAGG**ACAAAC). To generate point mutations in the *comGC* plasmid, DNA was isolated and a vector-PCR was used. Primer-pairs were designed back-to back with a size of 20 bp each, including the mutation at the beginning of the forward primer. Afterwards the DNA was de-phosphorylated, ligated, and in a further step template DNA was removed by *Dpn*I. The plasmid was transferred into chemically competent *E*. *coli* DH5α. Competent PY79 cells were transformed with plasmid DNA and integration at the *amyE*-site was verified by an amylase assay on starch agar. To this end, plates were covered with 5 ml of Iodine-potassium iodide solution (Carl Roth, Germany) and incubated for 10 min at room temperature. Each time the assay was performed, a positive and a negative control were included. List of strains see S3 Table.

To investigate point mutations at the original locus of *comGC*, the vector *pMAD* [42] was used. For generation of the PCR products and the point mutation, Gibson-Assembly and Q5-site directed mutagenesis was used. Primers were designed with the NEB primer design tool (New England Biolabs, USA).

### Transformation of *B. subtilis* & assays of transformation frequency

Freshly restreaked strains on LB-Agar plates were inoculated as a 3 ml LB-overnight culture, which was grown at 37°C and 200 rpm supplemented with inducing agents and antibiotics. The following day, a culture was inoculated to an OD_600_ of 0.08, and grown at 200 rpm at 37°C until the culture reached an OD_600_ of 1.5 in MC-media (see growth). For transformation 0.5–1.5 μg of chromosomal or plasmid DNA was added to the cells and incubated for 1–2 h at 37°C and 200 rpm. Transformants were obtained after an incubation time at 30 degrees for 48 h.

For transformation frequency assays, 0.5 ml of competent cells were incubated with 0.5 μg of chromosomal DNA, to have a final concentration of 1 μg/ml for 1 h at 37°C and 200 rpm. A serial dilution was made, and 10^−1^, 10^−2^ or 10^−3^ dilutions were plated on LB- Agar plates with respective antibiotics to obtain colony forming units (CFU). A 10^−6^ dilution was used to obtain the number of viable cells. By dividing the CFU/ml by the number of viable cells/ml/μg DNA, efficiency was calculated. For each strain a technical and biological duplicate was performed. Visualisation of data was generated with GraphPad Prism6 (GraphPad Software, San Diego, California, USA). To test the influence of Alexa Fluor 488 C5 Maleimide (AF488-C5 maleimide) on the transformation activity of the strains, cells were treated with the stain before addition of chromosomal DNA.

### Fluorescence staining of DNA

DNA was fluorescently labelled according to a modified protocol of Boonstra *et al*. [43]. A PCR product containing an Erythromycin resistance cassette flanked by homologous *B*. *subtilis* regions with a size of 2300 bp was employed for staining by using the primers pMB013 and pMB014 [31]. 5-[3-aminoallyl]-2’-deoxyuridine-5’-triphosphate (aminoallyl-dUTP, Thermo Scientific) was incorporated into DNA by a PCR using a DreamTaq DNA Polymerase (Thermo Scientific) and template DNA was removed by using *Dpn*I for 1.5 h at 37°C. The PCR product was purified and eluted in 50 μl of 0.1 M NaHCO_3_ solution at a pH 9. DNA was stained by using Dylight562 NHS Ester (Thermo Scientific), which binds to aminoallyl-dUTP. For reaction with the stain, an aminoallyl-ester with 10x excess of dye was added to the PCR product. In a further step the PCR product was labelled for 3h at 25°C in the dark. The amount of dye was calculated by:

$$\frac{m[DNA]}{M[DNA]}\times 10\times M[fluorophor]=m[fluorophor]$$ (1)

m = mass, M = molecular mass

The DNA was purified by using a PCR purification kit (Quiagen) with an additional washing step. Labelling of the PCR product was checked by fluorescence signal with the Biomolecular Imager (Typhoon, Amersham) of a 1% agarose-gel. Labelled PCR-products were stored at -20°C under exclusion of light.

### Staining of pilus structures

Competent cells were centrifuged with an Eppendorf centrifuge 5424 at 8000 rpm for 2 minutes, washed with 1 x PBS (pH 7) and subsequently labelled with 25 μg/ml AF488 C5 Maleimide (Invitrogen) for 10 min in the dark followed by at least two additional washing steps. The pellet was resuspended in supernatant from cells grown to competence (stationary phase from OD_600_ ~ 2).

### Epifluorescence microscopy

Cells grown to competence by incubation at 37°C and 200 rpm to an OD_600_ of 1.5 were transferred onto 1% agarose pads (w/v) containing conditioned medium, generated by sandwiching 100 μl of melted agarose between two coverslips (12 mm, Menzel). 3 μl of the culture were spotted onto a round coverslip (25 mm, Marienfeld). For wide field image acquisition, a Zeiss Observer A1 microscope (Carl Zeiss, Oberkochen, Germany) with an oil immersion objective (100× magnification, 1.45 numerical aperture, alpha Plan-FLUAR; Carl Zeiss, Germany) was used with a charge-coupled-device (CCD) camera (CoolSNAP EZ; Photometrics, AZ, USA) and an HXP 120 metal halide fluorescence illumination. A YFP filter set (ET500/20 EX T515lp ET535/30 EM) was used for epifluorescence or 488 nm laser excitation. Samples were illuminated for 0.5 to 2 s, dependent on signal intensity, at the midcell plane. Final editing of images was done with ImageJ2/ FIJI 1.52 [44].

### Single-molecule tracking (SMT)

Cells grown to competence were prepared with AF488-C5 maleimide (see staining of pilus structures). In case of incubation with chromosomal DNA, cells were incubated with 20 μg/ml chromosomal DNA (PY79) for 20 minutes as described [29]. Slides and samples for microscopy were prepared as described in Epifluorescence microscopy. For Single molecule tracking (SMT) an inverted microscope (Nikon Ti Eclipse) was used, with a 514 nm laser diode (100 mW, Omicron Laser) with an A = 1.49 objective and an EMCCD camera (Hamamatsu), using an integrated autofocus on the microscope. ImageJ2/ FIJI 1.52 [45] was used for movie cutting and processing. Movies were cut to reach a single molecule level by removing the initial bleaching steps, until the bleaching curve had a remaining decline of less than 10%. Cell meshes were set with oufti [46] and tracks were detected with UTrack [47]. In a further step tracks were analysed with the SMTracker software 2.1 [48]. Diffusion constants and fraction sizes were calculated by a squared displacement analysis (SQD). The SQD is the cumulative probability function of squared displacements.

### SIM Microscopy

Structured illumination microscopy was used for generating 3D reconstructions of cells. Images were acquired with the ZEISS ELYRA PS.1 (Laser lines 561nm/ 488nm; ANDOR iXon EMCCD; ZEISS objective: alpha Plan-Apochromat 100x/NA 1.46). Reconstructions were generated with the software ZEN-Black. Final editing and 3D-reconstructions of images was done by ImageJ2/ FIJI 1.52 [44].

### Western blotting

50 ml of competent cells were harvested and resuspended in appropriate volume of PBS buffer to reach an optical density OD_600_ of 3. Buffer was supplemented with lysozyme (3 mg/ml) to efficiently lyse the cells, before they were lysed with a sonicator. Lysed cells were incubated with varying concentrations of DTT (50 mM, 100 mM, 150 mM) for ten minutes at room temperature (if indicated) to break disulphide-bonds. Samples were incubated with SDS-loading buffer (40% glycerol [v/v], 500 mM Tris-HCL [pH 6.8], 8% SDS (w/v), 20% β-mecaptoethanol (v/v), bromphenol blue 10 mg/ml) for 1h at 60°C. 20 μl of each strain was loaded on a 12% acrylamide-gel and separated via gel-electrophoresis in SDS-running-buffer (10xSDS-RB: 3.03% [w/v] Tris, 14.4% [w/v] glycine, 1% [w/v] SDS). Acrylamide gel was blotted on an nitrocellulose membrane soaked in transfer-buffer (48 mM Tris, 39 mM glycine, 1.3 mM SDS pH 9.8) sandwiched between three layers of transfer-buffer soaked Whatman-paper and blotted for 38 minutes at constant voltage (15 V). Membrane was further blocked with 5% skimmed milk powder in PBS buffer (pH 7) for 1h, before overnight incubation of the first antibody with a dilution of 1:500 of Anti-ComGC in 5% milk with PBST (8.1 mM Na_2_HPO_4_ x 2H_2_O, 21.74 mM NaH_2_PO_4_ x H_2_O, 100 mM NaCl, and 0.1% Tween 20). To sequester accessory antibody, the blot was further washed in PBST buffer three times for 5 minutes. Subsequently, an incubation with the second antibody (Anti-rabbit lGG, Sigma Aldrich) to a concentration of 1:10000 in 5% milk with PBST occurred, before the three repetitive washing steps. The blot was detected with Western HRP substrate (Millipore).

### SPP1 phage transduction

Transfer of chromosomal DNA (PY79, *∆comGA)* was performed by SPP1-mediated phage transduction to the recipient strain PY79, *amyE*::*comGC*^*CYS*^. Phage lysates were prepared by mixing SPP1 phage stocks (in dilutions) with a bacterial culture. Infected culture was incubated for 15 minutes at 37°C. Sample was supplemented with TY soft agar (0.3%) and plated on a 1.5% TY-plate. Plaque formation can be detected after 16 h of incubation at 30°C. TY-medium was added to the plate and phages were harvested by collecting the soft agar/ liquid medium mixture. Sample was centrifuged and supernatant was filtered through a 0.2 μm pore size syringe filter. For transfection, 1 ml of the respective stationary grown *B*. *subtilis* recipient strain was inoculated with 30 μl of the phage lysate in 10 ml TY media (5 g/l NaCl, 5 g/l yeast extract, 10 g/l tryptone, 1 mM MgSO_4_, 10 μM MnSO_4_). The mixture was incubated on a shaking platform for 30 minutes at room temperature, followed by a centrifugation step to sediment the cells. The pellet was resuspended in approximately 100 μl of the remaining supernatant and plated on antibiotic containing LB agar plates supplemented with 10 mM sodium citrate to prevent phage re-infection.

## Results

### ComGC can be modified into a cysteine-carrying variant retaining transforming activity

**Fig 1:**
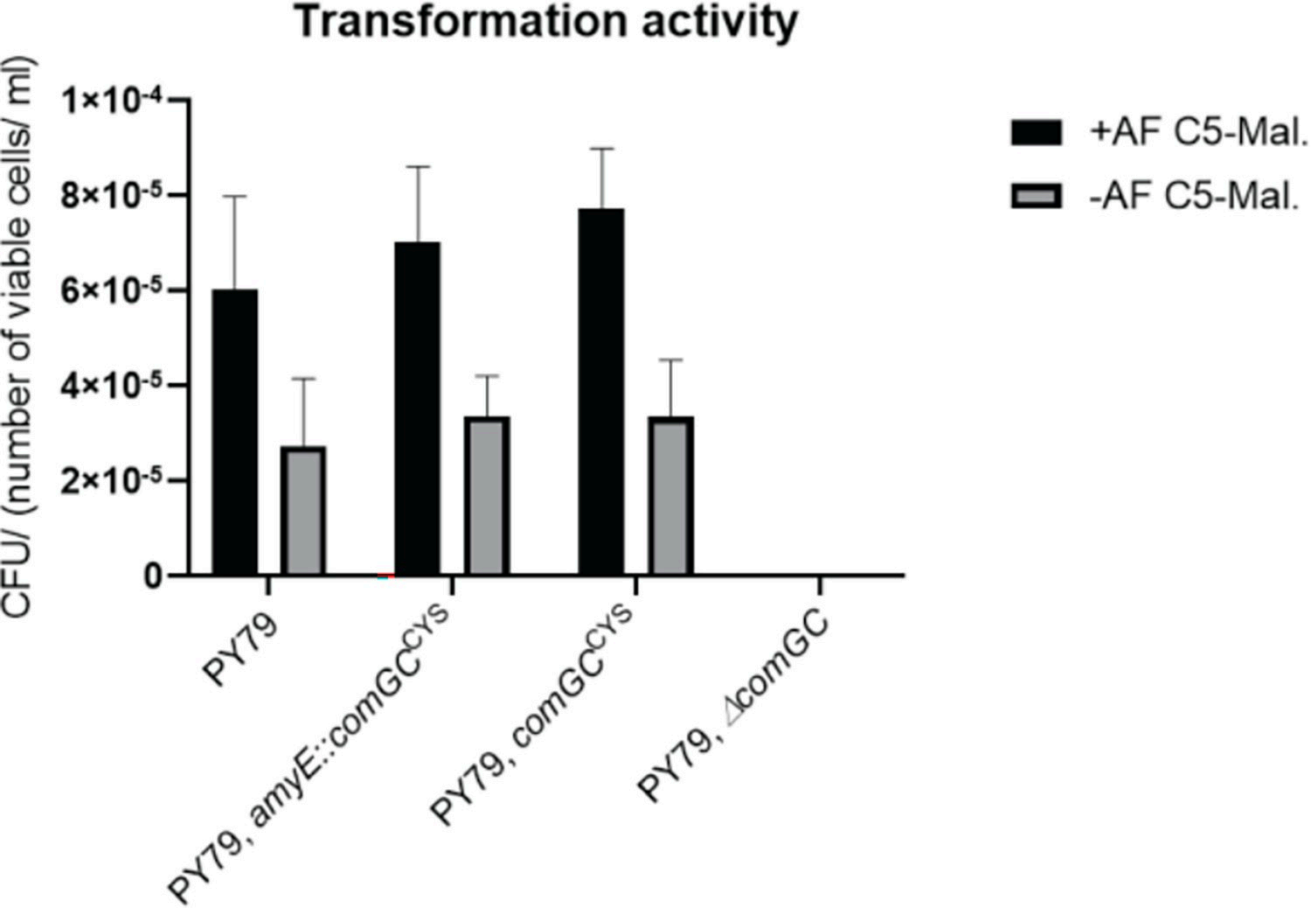
Transformation frequencies of strains encoding ComGC from the amyE locus and mutations. The bar plot indicates the CFU (colony forming unit) defined as the number of viable cells per millilitre per microgram added chromosomal DNA. Experiments were done in technical and biological duplicates. Error bars indicate the standard deviations. AF C5-Mal: AF-488 C5-maleimide stain.

We pursued the strategy of cysteine-labelling to visualize a possible pseudopilus or pilus structure in *B*. *subtilis* cells grown to competence. We mutated several amino acids of ectopically integrated ComGC to cysteine residues, and integrated these into the *amyE* site on the chromosome, driven by an IPTG-inducible promoter. We found a change at position 71 to cysteine to retain transformation activity, even when cells were incubated with a fluorescent maleimide stain to label ComGC (Fig 1). As expected we were not able to obtain transformants in a *∆comGC* deletion strain, which we used as a negative control [22]. In a next step, we integrated the corresponding codon for the 71C amino acid change into the original *comGC* gene, and tested for transformability. The mutation did not lead to a reduction in transformation activity, yielding 3.35 x 10^−5^ +/-1.19x10^-5^ CFU/(number of viable cells/ ml) (Fig 1), indicating that the point mutation does not have an effect on the functionality of ComGC. We were surprised that the Alexa Fluor488 C5 Maleimide (termed “AF488-C5 maleimide from here on)—staining procedure generally increased transformation efficiency of all constructs tested, independent of the presence or absence of the ComGC^CYS^ variant (Fig 1). While the finding shows that staining did not have a negative effect on the functionality of ComGC, we can not offer a conclusive explanation for the effect; possibly the staining procedure alters physical features of pili strengthening interaction with DNA.

Because we anticipated that a mixture of wild type ComGC and AF 488 C5-maleimide-labelled ComGC might be beneficial for pilus formation, based on the idea that too many labels within a pilus might hamper dynamics and thus imaging, we expressed the cysteine variant from the amylase locus, using mild IPTG induction. We found comparable transformation efficiencies like wild type cells (Fig 1). As a control for the following experiments, we created a strain that ectopically expresses wild type ComGC from the *amyE* locus. For this strain, we detected higher numbers of transformants with an activity of 1.13 x 10^−4^ ± 1.03 x 10^−4^, or 2.16 x 10^−4^ ± 1.84 x 10^−4^ after treatment with AF 488 C5-maleimide (missing in Fig 1 because of the high standard deviation). Because of the high standard deviation, we cannot conclusively state that higher amounts of ComGC generally increase efficiency. In spite of the inexplicable effects of increased transformation via staining and ectopic ComGC expression, the experiments suggest that a change of serine at position 71 to cysteine leads to sufficient activity of ComGC to study its *in vivo* localization.

To investigate expression levels of ComGC^CYS^ variants we performed immunoblot analyses with specific antiserum raised against ComGC [28] (S1 Fig). Unspecific binding was observed for the anti-serum in a *comGC* deletion strain (PY79, ∆*comGC*). In the tested strains (PY79) as well as in PY79, *amyE*::*comGC*^*CYS*^, additional signals were visible at ~12 and ~20 kDa. The lower band likely contains ComGC in an unprocessed and processed form (mediated by the prepilin peptidase ComC), and a strong dimeric form. The latter is in agreement with our preliminary data suggesting dimer formation for ComGC *in vitro*. ComGC levels did not alter considerably upon IPTG induction of ComGC^CYS^ (S1 Fig).

### *Bacillus* cells grown to competence carry a true pilus on their surface

**Fig 2:**
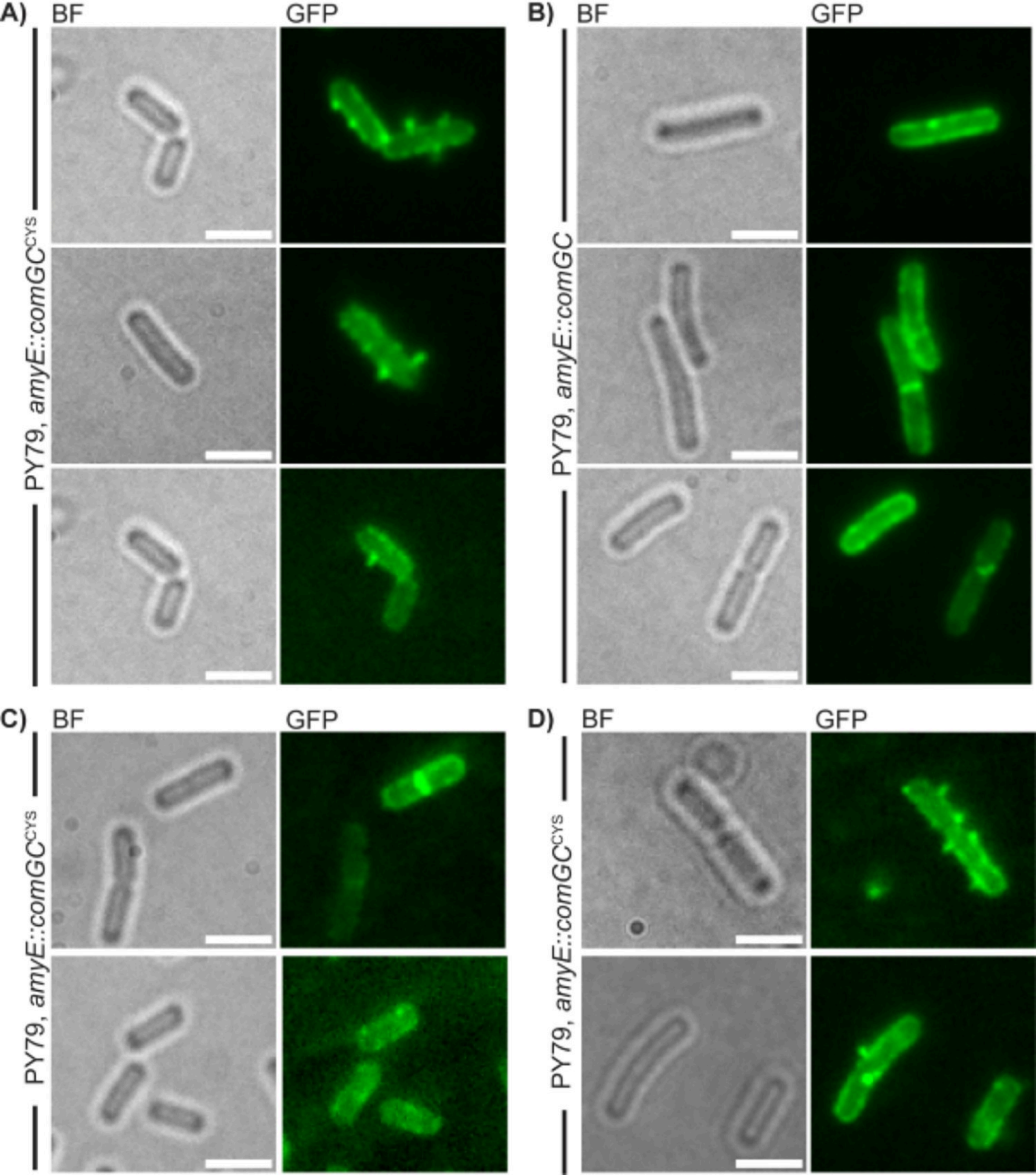
Epifluorescence microscopy of AF488 C5-maleimide stained cells of mutated cells (PY79 *amyE*::*comGC*^*CYS*^*)* and cells (PY79 *amyE*::*comGC)* encoding ComGC at the *amyE* locus. With 0.5 mM IPTG induction (panel A and B), without IPTG induction (panel C) and with low (0.01 mM) concentrations of IPTG (panel D). Left images of a panel show bright field images (BF-channel), right panels show epifluorescence pictures (GFP-channel, i.e. imaging of 488 nm fluorescence excitation). Scale bars represent 2 μm.

When cells expressing ComGC from an ectopic site were fluorescently labelled by treating the cells with AF488 C5-maleimide, filamentous structures emanating from the cell surface were observed (Fig 2A), indicating that a pilus-like structure can be visualized in *B*. *subtilis* cells. Filamentous structures were observed near the cell poles (Fig 2A, mid panel), and at different positions along the lateral side of cells (Fig 2A, lower panel); often, more than one structure per cell was visible (Fig 2B upper panel). The structures were not seen in the negative control, which expresses ComGC from the *amyE* locus of competent cells (Fig 2B). Instead, only staining of the membrane and of the septum was detectable. Counting of 385 of such cells revealed that due to the lack of a surface-exposed cysteine residue, fluorescent pilus structures could not be visualized.

Contrarily, in cells expressing ComGC^CYS^, 64 cells showed pilus structures out of 318 analyzed cells, which is in accordance with the literature value of 10–20% of cells inducing the state of competence [19]. The formation of the structures was dependent on IPTG, cells incubated without any IPTG did not show any pili (Fig 2C). To ensure that the formation of the pilus structures is not based on overexpression, we used a low concentration of IPTG (0.01 mM). Even with low induction we were able to see pili (Fig 2D), which supports the assumption that the formation was caused by incorporation of ComGC^CYS^ into existing ComGC-based pili. We wanted to know if pilus formation is dependent on external DNA. For this, we incubated a ComGC^CYS^ AF488 C5-maleimide stained strain with chromosomal DNA and quantified the number of pili in these cells by using Epifluorescence microscopy. By counting ~200 cells we were not able to observe any striking change in pilus quantity by treatment with chromosomal DNA, which lead to a slight decrease of 1.2 fold in formation of pilus structures, suggesting that pilus formation is not depending on the availability of DNA in a culture.

Of note, we likely underestimated the number of pili, because we did not count fluorescent foci, which occasionally were also observed in cells (< 1%) lacking the ComGC cysteine-variant (Fig 2B), although foci in the ComGC^CYS^ strain might represent very short pili. To further address this point, we analysed ComGC^CYS^ structures in cells lacking ComGA, the assembly ATPase crucial for pilus formation [49]. For this purpose, a *comGA* deletion was transduced into the ComGC^CYS^ variant strain. No exposed surface structures could be observed, and foci were found in less than 1% of cells (S2 Fig). This control confirms the role of ComGA as being essential for pilus extension [49], and suggests that foci can arise as part of background staining.

**Fig 3:**
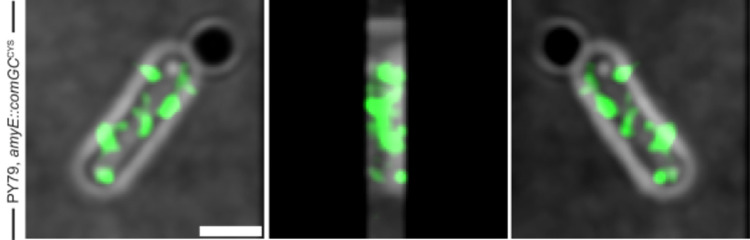
3D-reconstructions of Structured illumination microscopy of AF488 C5-maleimide-stained cells encoding ComGC^CYS^ from the *amyE-*locus. Left panel shows 0°C, middle panel 180° and right panel 270°. Corresponding S1 movie. Scale bars represent 2 μm.

We used 3D super-resolution Structured Illumination Microscopy (SIM), reaching a resolution of 125 x 125 nm in x and y direction and ~250 nm in z-direction, to generate 3D reconstructions of cells. With this technology, it is feasible to determine if a cell envelope-spanning machinery is exposed from the surface of *B*. *subtilis*. Z-stacks of stained cells were obtained, and signal was seen at sites emanating away from the cell surface (Fig 3, S1 movie). S2 movie displays Z-stacks of AF488 C5-maleimide stained cells. Staining of cells expressing ComGC^CYS^ from the original gene locus did not show structures that could be well-resolved. We favour the view that pili can be visualized best when a mixture of native (non-labelled) and cysteine-mutant (labelled) ComGC is expressed in the cells. We further used 2D SIM to measure the length of pili, which was 505 ± 155 nm (n = 35) under full induction (0.5 mM) of the hyperspank promoter. To ensure that length is not influenced by overexpression of labelled ComGC, we also measured pilus length using low (0.01 mM) IPTG induction. Pilus length was 539 +/- 40 nm (n = 40), showing that length does not correlate with induction levels. Thus, keeping in mind the caveat that we used a merodiploid strain to measure average pilus length, we believe it is safe to argue that *B*. *subtilis* pili are considerably shorter than those observed for Gram-negative bacteria [34, 35]. These findings support the idea that DNA uptake through the cell wall in *B*. *subtilis* is not restricted to the cell poles, as was suggested by earlier work using immunofluorescence and FlaSH-tag staining [28], but occurs all over the cell surface, because competence pili were detected at many places on the cell surface.

### Pili are dynamic structures that interact with external DNA

**Fig 4:**
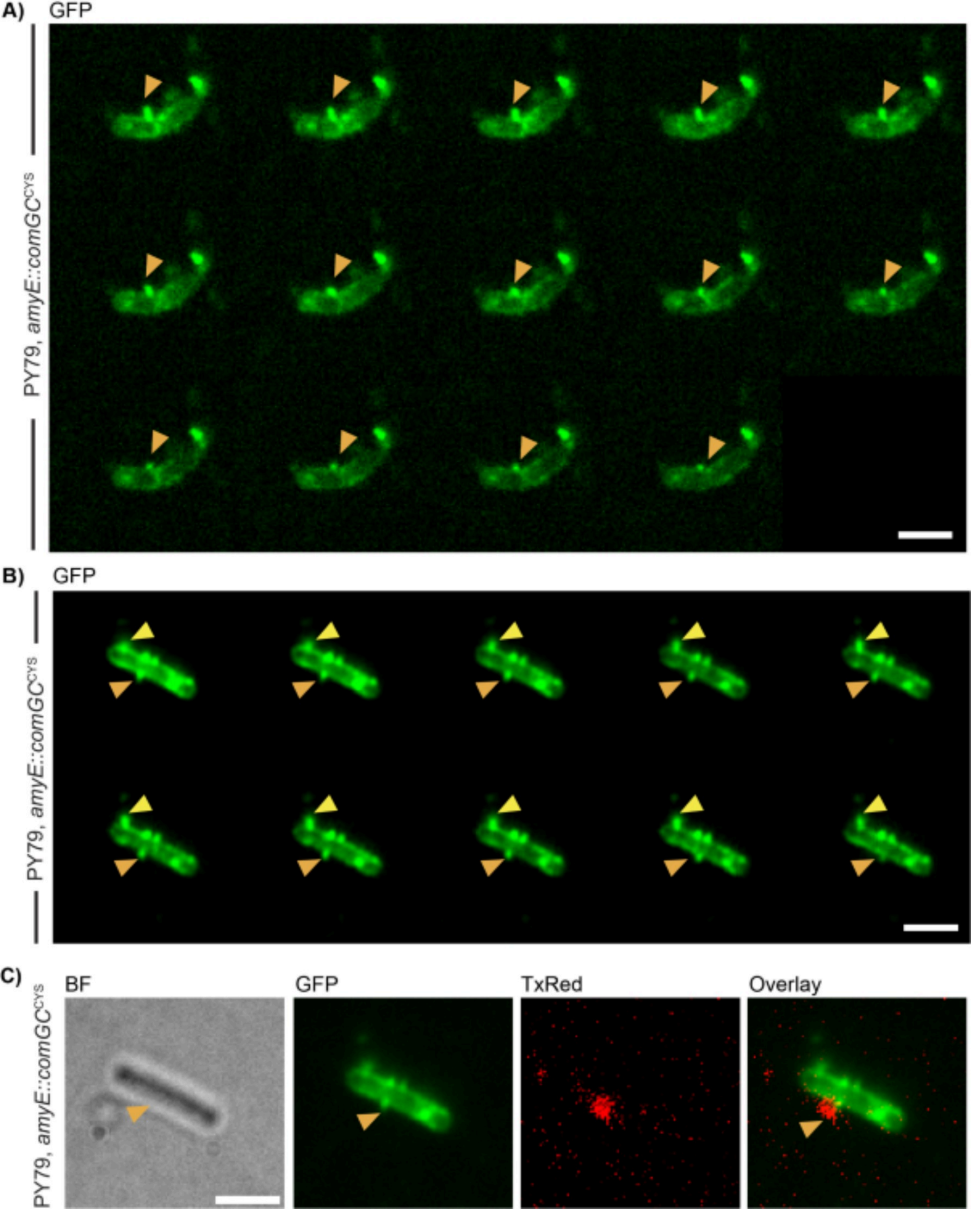
Dynamics of AF488 C5-maleimide-stained competence pili. A+B) Images of time-laps microscopy were acquired every 20 s. Yellow arrow indicates pilus formation, orange pilus retraction. Movies were acquired in the GFP-channel. C) Colocalization of fluorescently labelled DNA with C5 Maleimide-stained competence pili in BF-, GFP, and TxRed-channel. Scale bars 2 μm.

Competence pili as well as pili used for surface-based motility are known for dynamic extension/retraction periods, in which pili grow by addition of pilins to the base of the pilus, or by detachment of pilin subunits from the base, and their presumed diffusion back into the cell membrane. We tested if *B*. *subtilis* pili show such time-dependent remodelling, using 20 seconds time lapse experiments. Fig 4A shows an example of a cell displaying pili; the orange triangle indicates a pilus that is retracting (S3 movie). Fig 4B highlights a cell, in which a pilus that is extending (yellow triangle), and a pilus indicated by the orange triangle retracts within the last two time intervals (see S4 movie). Of note, we did not observe considerable lateral motion of pili along the cell surface. These data suggest that pili are stably anchored in the cell membrane, and their lateral motion is restricted through the rigid cell wall, such that they remain static on a scale of 3 to 4 minutes. A significant number displays extension/retraction dynamics in a second-time scale, expected for a structure that pulls external DNA through the cell wall.

To test if an interaction between DNA added to the culture and competence pili can be observed, we used fluorescently labelled DNA in strains expressing ComGC^CYS^. Labelled DNA was checked for fluorescence signal with a biomolecular imager, which showed fluorescence signal at 2300 bp corresponding to the size of the amplified PCR product, including an erythromycin resistance cassette and homologous regions of the *B*. *subtilis* chromosome [31]. Fluorescently labelled DNA was incubated with AF488 C5-maleimide-stained cells that were treated with DNase in a further step to ensure that no additional, exogenous DNA was present in the culture. Indeed, we were able to detect fluorescence signal for the labelled DNA that colocalized with pilus structures (Fig 4C). This event was seen in few cells, suggesting that colocalization of taken up DNA by a pilus structure is a rare event, or, alternatively, that the assay is not well suited to visualize pilus/DNA interaction. Of note, fluorescently labeled DNA yielded a normal number of transformants, comparable to non-labelled DNA [31, 43].

### ComGC^CYS^ is diffusing along the membrane in SMT

Pilin subunits of pili are expected to diffuse through the cell membrane, and to assemble into a polymeric structure. Because we are not aware that dynamics of pilin have been visualized at a single molecule level, we set out to obtain more information on the dynamics of competence pili and performed single molecule tracking experiments with ComGC^CYS^, expressed at very low levels from the *amyE* site. Because transformation efficiency was high in this strain, and because it is reasonable to assume that ComGC^CYS^ mixes with wild type ComGC and forms joint pili, we tracked ComGC under these conditions, which appear to most closely resemble native conditions. In other words, because ComGC^CYS^ is visibly incorporated into pilus structures, it should serve as good proxy to ComGC dynamics *in vivo*. SMT allows to follow the motion of freely diffusive molecules in living bacteria with a resolution of 40 nm and less. For tracking of single molecules, we used a 30 ms stream acquisition. Non-interrupted tracks of at least five steps were used for data analysis. SMTracker 2.0 was used for data analysis of single molecule tracks that were generated with U-track [47].

**Fig 5:**
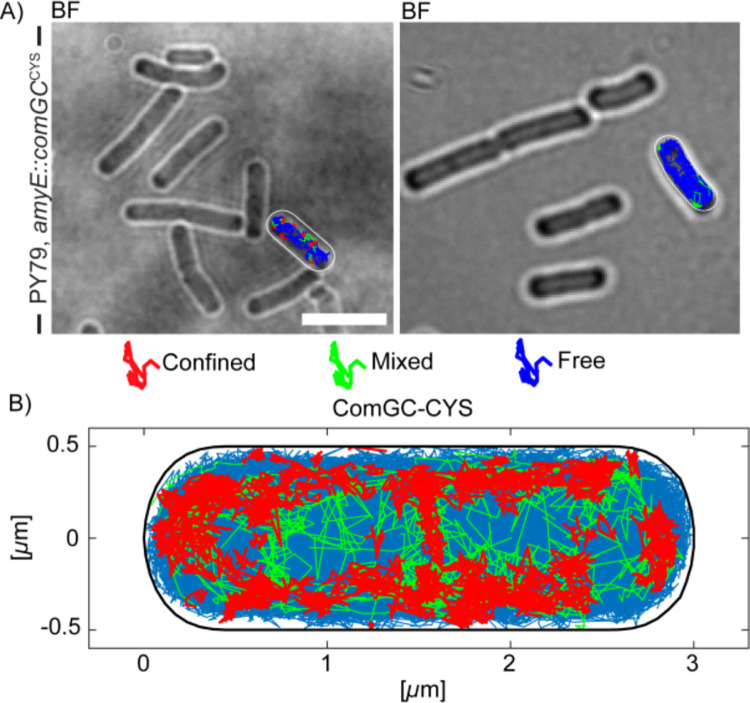
Localization of Single molecule tracks of ComGC^CYS^ treated with AF488-C5 maleimide. A) Two examples of overlays of Confinement maps in representative cells, showing confined (red) or mobile (blue) tracks, or those showing transitions (green). B) Confinement map of confined tracks projected into a standardized *B*. *subtilis* cell. Confinement radius is 108.7 nm, which corresponds to 3 times the localization error. Confined tracks are indicated in red, green tracks show tracks from a confined to a more mobile movement and *vice versa*, while blue tracks are defined as mobile, moving out of the confinement radius. Scale bar represents 2 μm.

At first, we localised ComGC^CYS^ molecule by projection of tracks into a normalized (medium-size) *B*. *subtilis* cell with an average size of 3 x 1 μm. Red tracks in Fig 5A represent molecules moving in confined motion, within an area of 108.7 nm in diameter, in cells grown to competence. The confined area corresponds to 3 times the localization error determined from mean squared displacement (MSD) analyses. Confined motion was restricted to the cell membrane, and the septum at mid-cell, as expected for a membrane protein (Fig 5B). Confined tracks likely represent ComGC^CYS^ molecules engaged in pilus formation. Freely diffusing molecules are shown in blue; note that the cell is over-saturated with these tracks in order to obtain a sufficient number of confined tracks. Green tracks represent molecules showing transitions between diffusive and confined motion, and largely overlap with areas of confined motion.

**Fig 6:**
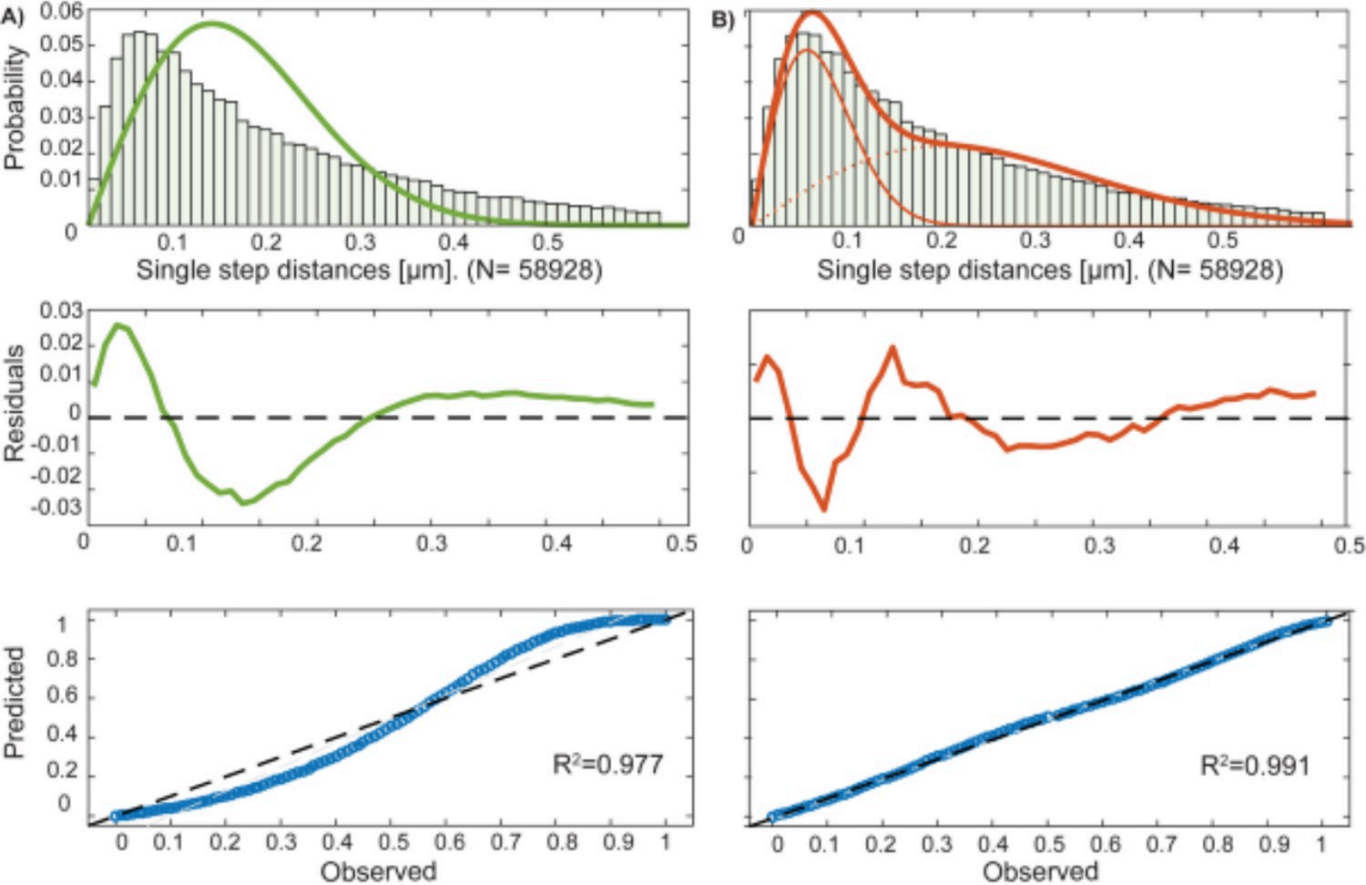
Squared Displacemend analysis (SQD) of ComGC^CYS^ cells treated with AF488 C5-maleimide in *B*. *subtilis* cells grown to competence. A and B (upper panel) show Rayleigh fits of the generated data of one and two populations. The plot shows the frequency of diffusion constants depending on the diffusion coefficient of the tracks in a histogram. The probability R^2^ is annotated for each histogram, a two population fit is shown in B, which better describes the data as in A. Lower Panels of A and B show quantile–quantile plots for one (A) and two populations (B) which illustrate how well measured (dashed line) and the modelled (blue line) data fit together.

**Fig 7:**
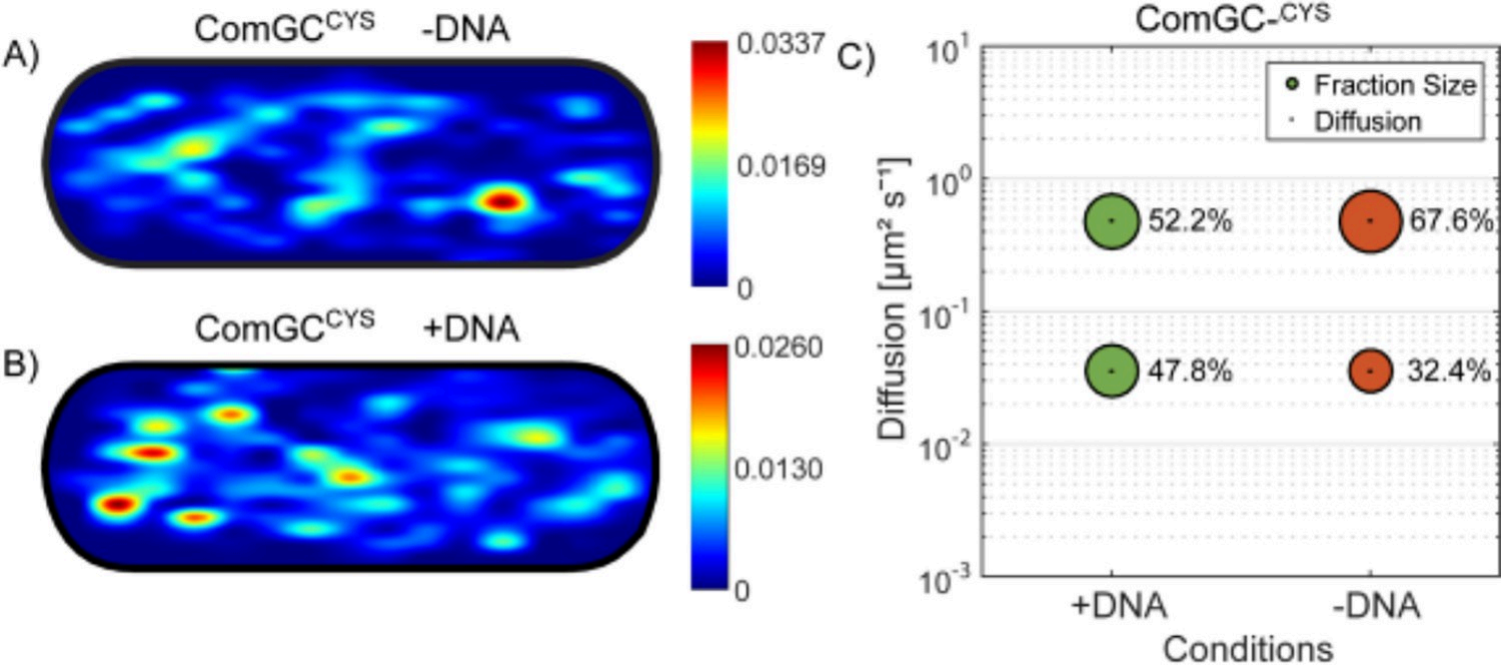
Response of pilus dynamics after addition of DNA. Panels A) and B) show heat maps of confined tracks of (AF488 C5-maleimide labelled) ComGC^CYS^ in cells projected into a standardized *B*. *subtilis* cell. Confinement radius was 108.7 nm. Colour code on the right indicates intensity of signals. “- DNA” and “+ DNA” indicates untreated cells or cells treated with DNA for 10 minutes. C) Bubble plot shows diffusion constants [μm^2^/s] the fraction sizes of populations by setting a simultaneous diffusion constant which is shown in correspondence. Errors correspond to the 95% confidence intervals which are given by “confint” matlab function by using its values that result from the fit. +DNA and -DNA indicates cells which were treated with DNA for 10 minutes.

Jump distance (JD) analysis was used to obtain more detailed information about the diffusion constants and fraction sizes of ComGC^CYS^. JD analysis describes the Eucledian distance between consecutive detections [48]. A nonlinear regression fit was used to describe the data (Rayleigh fit). By using one Rayleigh fit, the generated data were not well represented (Fig 6A, upper panel). Two Rayleigh fits had to be used to describe the data sufficiently well (Fig 6B, upper panel). Middle panels show the difference between fitted data and modelled data based on Brownian diffusion. Please note the different Y-axis dimensions, showing that differences were much smaller when using two Rayleigh fits (Fig 6B). Lower panels present overall fitting of data with modelled data, which shows that two fits leave very little deviation (Fig 6B), compared with the single fit (Fig 6A). These analyses suggest that two populations with distinct diffusion constants exist for ComGC^CYS^ *in vivo*. Using JD data, diffusion constants and fraction sizes of the two populations were determined. Fig 7B visualizes the data shown in Fig 6B: the size of the bubbles corresponds to the relative size of the population and the height along the Y-axis to the diffusion constant of the protein. The slow population (“static fraction”, Pop_1_) had a size of around 32% of ComGC^CYS^ molecules, with a diffusion constant (D_1_) of 0.042 ± 0.001 μm^2^/s, while the faster and larger population (Pop_2_) with 68% of ComGC^CYS^ had a diffusion constant (D_2_) of 0.51 ± 0.001 μm^2^/s (Table 1). The latter is well within the range of diffusion constants obtained for other freely diffusive membrane proteins in *B*. *subtilis* [50], the prior with a rather static structure. Treatment of PY79 *amyE*::*comGC* cells with Alexa-Fluor C5-maleimide showed unspecific binding of the stain (Fig 2B). To control for ComGC^CYS^ tracks due to unspecific binding of the stain, we performed single-molecule tracking of PY79 *amyE*::*comGC* with and without staining. S3 Fig shows than less than 10% tracks were monitored in cells lacking staining and/or the cysteine exchange; non-specific tracks were also considerably more slow than those observed for stained ComGC^CYS^ (S3 Fig, bubble blots). These data suggest that about a third of ComGC molecules are statically engaged in pili, while two third are diffusive within the membrane, able to be recruited into pilus structures.

**Table 1:** Single molecule tracking of ComGC^CYS^ with Alexa-Fluor C5-maleimide treatment, with/ and without addition of DNA with an incubation time of 10 minutes.

	PY79 *amyE*::*comGC*^*CYS*^*—*DNA	PY79 *amyE*::*comGC*^*CYS*^ *+* DNA
Pop_1_ [%]	32.4	47.3 ± 0.005
Pop_2_ [%]	67.6	52.7 ± 0.005
D_1_ [μm^2^/s]	0.04 ± 0.001	0.038 ± 0
D_2_ [μm^2^/s]	0.51 ± 0.001	0.22 ± 0.003

### The static fraction of ComGC^CYS^ increases after addition of chromosomal DNA

We wished to address the question if competence pili that interact with DNA show altered dynamics. For this we tested different incubation times for cells expressing ComGC^CYS^ with and without exogenously added chromosomal DNA. Tracking of ComGC^CYS^ after 20 minutes incubation time with DNA yielded similar sizes of populations and of the diffusion constants (S4 Fig). Considering that possibly, most of the added DNA is already taken up in 20 minutes and located either in the periplasm or in the cytosol, we used a shorter incubation time of 10 minutes for following experiments. Also, we ensured that only cells with visible pilus structures were analysed. For this, an epifluorescence image was taken before tracking and only cells with visible exposed surface structures were used for further analysis. Here, the static fraction of ComGC^CYS^ showed a significant shift of its size from 32.4% to 47.8% (Fig 7C). Concomitantly, the diffusion constant of the static fraction somewhat decreased from 0.042 to 0.033 ± 0.001 μm^2^/s with addition of DNA, while that of the fast population only mildly decreased from 0.51 to 0.45 ± 0.001 μm^2^/s (Table 1).

Moreover, by projecting approximately 500 tracks into a normalized *B*. *subtilis* cell of 3 x 1 μm size and sorting the tracks which stayed within a confined radius of 108.7 nm we observed a change in the localisation pattern (Fig 7A). The degree of confined tracks at the cell periphery was visibly increased (note that *B*. *subtilis* cells are 0.7 μm wide), likely representing ComGC within active pili (Fig 7B), in agreement with the increase to 47% increase in the static population (Fig 7C). However, this change in confinement is not reflected in the number of pili visible by SIM or epifluorescence microscopy, which was only 1.2 fold higher in cells after addition of DNA. We interpret these findings to indicate stochastic, DNA-independent polymerization of competence pili, and an increased time ComGC spends in its polymerized form, suggesting a longer time for retraction due to bound DNA.

## Discussion

*B*. *subtilis* cells take up DNA into the cytosol at a single, and less often at both cell poles [26]. This localized transport through the ComEC channel leads to the assembly of recombination proteins at a single subcellular site, possibly facilitating search for homology on the chromosome of incoming DNA by a two-dimensional process [27]. It has been unclear if DNA is also taken up from the environment at a single polar site, which would decrease the probability of interaction with DNA, and if uptake occurs via a pseudopilus, i.e. a short structure that opens a gate through the wall, or via a true pilus, such as in *S*. *pneumoniae* cells, which can “fish” DNA along a longer distance away from cells [35].

It has long been known that *B*. *subtilis* cells contain an operon of pilin-like genes that plays an essential role in DNA uptake. The encoded ComG proteins have similarities to proteins forming Type IV-pili and type II-secretion systems of Gram-positive and Gram-negative bacteria [51]. Degradation of the cell wall relieves the essentiality of the operon, and biochemical experiments have shown that the major pilin, ComGC, forms a complex of a size of at least 400 to 1000 kDa, in conjunction with minor pilins [28, 51]. It was also shown that these proteins might facilitate the interaction of DNA with the DNA receptor [51].

Fusions of mCherry with ComGC showed no functionality in DNA uptake. By using immunofluorescence microscopy and a FlaSH-Tag, ComGC was shown to localize to the cell poles and the cell center [52]. Therefore, it was proposed that cells may contain just one active, assembled competence machinery and freely diffusive single competence proteins [52]. By using a pilus labelling method we show that indeed, *B*. *subtilis* cells possess a competence pilus, a machinery spanning through the cell envelope, which was detected in 20% of cells grown to competence. The length of the structures was analysed by structured illumination microscopy, a super-resolution method by which a resolution of down to 125 nm can be reached. Structures with an average of 500 nm length were measured, indicating a much shorter structure then reported for Gram-negative bacteria like *V*. *cholerae* [34].

**Fig 8:**
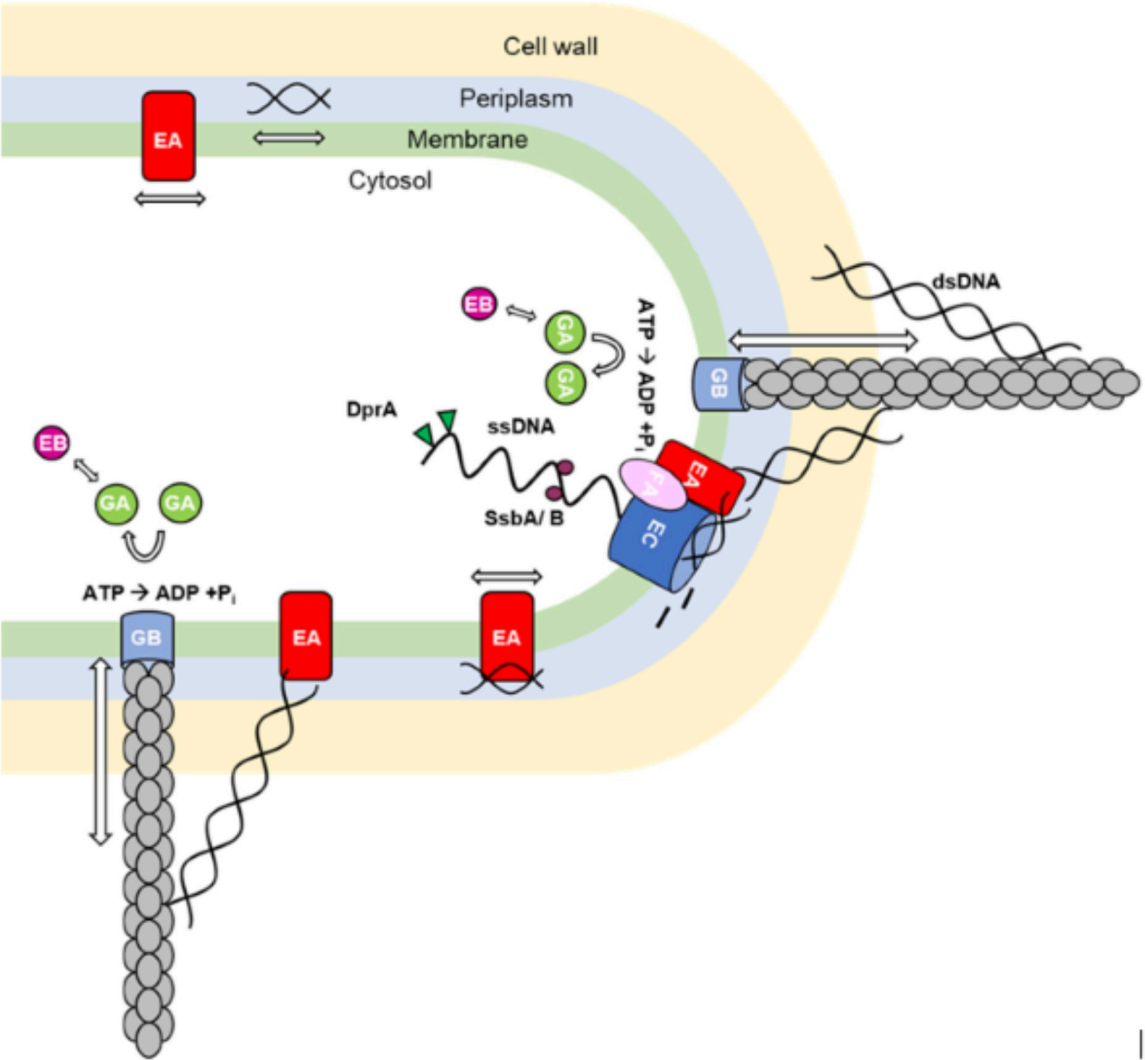
Model of DNA uptake in *B*. *subtilis* as a two-tier process. The periplasmatic uptake of double stranded (dsDNA) occurs over the whole cell surface via a true pilus structure, showing dynamic assembly and disassembly. The pilus structure is made out of minor (ComGD, ComGE, ComGG) and major pilins (ComGC) shown in grey. The anchor of the structure might be ComGB (light blue). Via pilus retraction, bound DNA will be pulled into the periplasm where it will bind to the DNA receptor ComEA (red), and diffuse throughout the periplasm. Once reaching the poles, it can be taken up by ComEC (dark blue). ComFA (violet) might provide the energy for the uptake. Direct passage through the pilus, via ComEA to ComEC, may be possible at the pole. ComEC processes double stranded (ds) DNA into single stranded (ss) DNA, which once inside the cytosol will be coated by single strand DNA binding proteins SsbA, SsbB (purple) and DprA (green). This is followed by an integration into the chromosome via dynamic RecA filaments.

Intriguingly, the *B*. *subtilis* competence machinery spanning the cell membrane as well as cytosolic proteins involved in DNA recombination have been shown to be located at one or both cell poles, suggesting that DNA uptake into the cytosol occurs at a single, specific site in competent cells [2, 4, 26–28, 30]. Our results reveal that the pilus in *B*. *subtilis* can be located at any site within the cell envelope, indicating that DNA uptake through the cell wall is not limited to the poles, in contrast to intracellular uptake through the cell membrane (Fig 8). Also, the presence of more than one competence pilus revealed that there can be more than one working structure for DNA uptake. This was already proposed by Boonstra et al. [43]: the authors showed that that fluorescently labelled DNA also was taken up at the cell center, and in fact even more often so than at the poles. Uptake at any site on the cell surface, using several pili, increases the efficiency of DNA uptake from the environment, while transport into the cytosol at a single, polar site, may organize RecA filaments that guide incoming DNA to the corresponding sites in the genome [27] (Fig 8). Likewise, storage of incoming DNA within the periplasm may increase transformation efficiency, and allow for slow transport of ssDNA into the cytosol during the concomitant possibility to rapid uptake of dsDNA through the cell wall.

**Fig 9:**
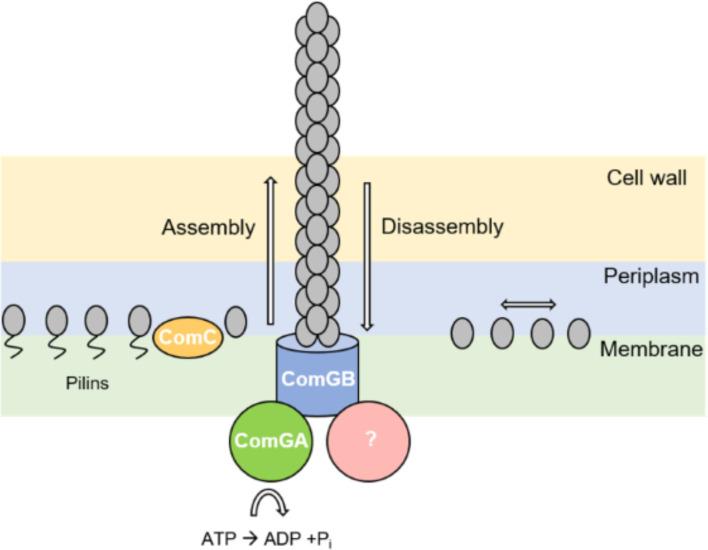
Model for extension and retraction of the competence pilus in *B*. *subtilis*. Pre-pilins are processed by the prepilin peptidase ComC (yellow) and are assembled into a competence pilus, for which ComGB (blue) might be the platform protein. The energy could be provided by assembly-ATPase ComGA (green), while a retraction factor, or retraction activity, is yet to be identified (purple). Processed ComGC (grey) diffuses within the membrane, while some of the molecules, after having assembled into a competence pilus, are positioned bound within the pilus polymer, represented the static single molecule fraction.

We also studied the dynamics of pilin ComGC at a single molecule level, for which we have not found earlier data in the literature. We found that as expected from models on pilus formation, a larger pool of ComGC freely diffuses within the cell membrane, while a third of the molecules is statically positioned at the membrane, likely bound within the pilus polymer (Fig 9). Incubation with chromosomal DNA resulted in a change in the dynamics of ComGC, by an increase of the population size for the static fraction. More pilus-bound ComGC could mean the formation of more pili in response to DNA uptake, but we did not observe this. Alternatively, our findings suggest that ComGC stays longer within a pilus structure that is pulling DNA across the cell wall than in an unbound pilus. Interestingly, we found an increase in the static ComGC population 10 minutes after addition of DNA (Fig 7B), but in contrast, a longer incubation time of 20 minutes for DNA uptake did not lead to a significant change in fraction sizes and diffusion constants (S3 Fig). We interpret these finding to suggest that probably most of the externally added DNA was already taken up within 20 minutes, indicating that the process happens efficiently within a relatively short time period.

Single molecule experiments have shown that *B*. *subtilis* cells pull on external DNA with a force characteristic of that typically reported for type IV-pilus retraction [53]. Retraction of pili containing labelled ComGC in our study occurred within seconds to few minutes, in good agreement with single molecule experiments. How might the pilus bind to dsDNA? Purified pilins from *Thermus thermophilus* and from *Streptococcus pneumoniae* have been shown to bind non-specifically to dsDNA [54, 55], possibly forming a tip-structure [56], while pilis from *Neisseria meningitidis* even bindwith higher affinity to DNA-uptake sequence (DUS) motif abundant in this species genome [57, 58]. Non-specific DNA binding to *B*. *subtilis* cells is reduced in *comGC* mutant cells [59], suggesting that the competence pilus also has affinity to dsDNA. We have not been able to find binding of purified, individual pilins to DNA, so it remains to be elucidated how DNA is attached to the pilus for its passage through the cell wall.

Taken together, our data are in favour of a model in which the DNA uptake is a two-tier process in which uptake into the periplasm, in which DNA can freely diffuse or be bound to ComEA [31, 38], occurs over the whole cell surface by one or several pili per cell, while cytosolic uptake across the cell membrane is limited to the poles [2], where binding of DNA recombination proteins guides incoming ssDNA onto the nucleoid(s) for homology search.

## Supporting information

S1 Movie 3D reconstruction of an AF488-C5 maleimide stained *B*. *subtilis* cell grown to competence expressing ComGC^CYS^ from an ectopic site on the chromosome. Movie played with a frame rate of 10 frames per seconds (fps).
(AVI)

S2 Movie Reconstruction of a Z-stacks of an AF488-C5 maleimide stained *B*. *subtilis* cell grown to competence expressing ComGC^CYS^. GIF is shown with 5 frames/s.
(GIF)

S3 Movie Time lapse (20 second intervals) of pilus structures of AF488-C5 maleimide stained *B*. *subtilis* cells grown to competence expressing ComGC^CYS^. (AVI)

S4 Movie Retraction and Extension of pilus structure of AF488-C5 maleimide stained *B*. *subtilis* cell. Time lapse (20 second intervals) of pilus structures of AF488-C5 maleimide stained *B*. *subtilis* cells grown to competence expressing ComGC^CYS^.
(GIF)

S1 Fig Immunoblot analysis of expression levels of ComGC in ComGC^CYS^ varaints. Cell lysates of cells grown to competence were used. Strains are indicated above the lanes. DTT at varying concentrations was used. Left blot shows immunoblot analysis using ComGC-antiserum (note that the serum detects two non-specific bands at about 50 kDa and 90 kDa, and two specific ComGC bands, with a band at 20 kDa possibly reflecting dimeric GomGC); right panel shows corresponding immunoblot analysis using EF-Tu-antiserum to ensure equal loading of cell extracts.
(TIF)

S2 Fig Epifluorescence microscopy of AF488 C5-maleimide stained cells of *comGA* mutated cells (PY79, *∆comGA*, *amyE*::*comGC*^*CYS*^*)* encoding *comGC* at the *amyE* locus with IPTG induction. Left images of a panel show bright field images (BF), right panels show epifluorescence pictures (GFP*-channel*). Scale bars represent 2 μm.
(TIF)

S3 Fig Single molecule tracking of ComGC and ComGC-CYS strains with and without treatment with AF488-C5 maleimide. Confinement maps of A) PY79, *amyE*::*comGC* with maleimide (A) and without (B), and PY79, *amyE*::*comGC*^CYS^ with (D) and without (C) treatment of AF488-C5 maleimide. Confinement radius 108.7 nm (E) Bubble plots show the different sizes for populations of ComGC and ComGC-CYS in cells treated with (+) and without (-) stain. Size of bubbles indicates the size of populations.
(TIF)

S4 Fig Single molecule tracking of ComGC^CYS^ with and without incubation of DNA. A) Heat maps of ComGC-CYS stained cells with AF488-C5 maleimide with and without incubation of chromosomal DNA (indicated above) in a standardized *B*. *subtilis* cell. Confinement radius was 108.7 nm. The colour code on the right indicates the intensity of the signal with presence of the protein. B) Bubble plot of ComGC-CYS with a simultaneous diffusion constant. Cells were treated as indicated (with) DNA for 20 minutes as indicated above. Note that population sizes and diffusion constants are different from Fig 7C, because simultaneous fits were used assuming the same optimal D for both conditions.
(TIF)

S1 Table Oligonucleotides used in this study. (DOCX)

S2 Table Plasmids used in this study. (DOCX)

S3 Table *E*. *coli* and *B*. *subtilis* strains used in this study. (DOCX)

S4 Table Single molecule tracking of ComGC^CYS^ with/ and without addition of DNA with an incubation time of 20 minutes. (DOCX)
