# Supplementary figures and images for: Uptake of environmental DNA in Bacillus subtilis occurs all over the cell surface through a dynamic pilus structure
Source: PLoS Genet. 2023 Oct 10;19(10):e1010696. doi: 10.1371/journal.pgen.1010696 (PMC10564135; doi:10.1371/journal.pgen.1010696)

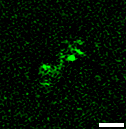

Supplement: S2 Movie — GIF is shown with 5 frames/s. (GIF) [file pgen.1010696.s002.gif]

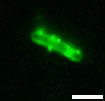

Supplement: S4 Movie — Time lapse (20 second intervals) of pilus structures of AF488-C5 maleimide stained B. subtilis cells grown to competence expressing ComGCCYS. (GIF) [file pgen.1010696.s004.gif]

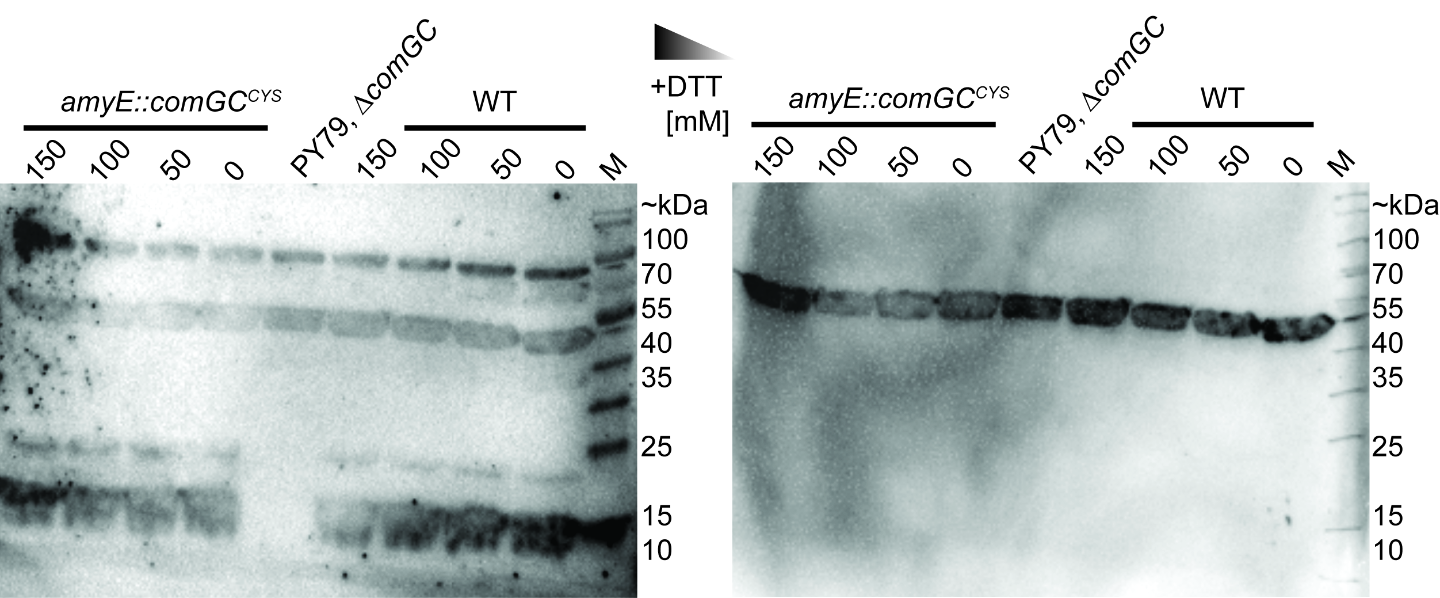

Supplement: S1 Fig — Cell lysates of cells grown to competence were used. Strains are indicated above the lanes. DTT at varying concentrations was used. Left blot shows immunoblot analysis using ComGC-antiserum (note that the serum detects two non-specific bands at about 50 kDa and 90 kDa, and two specific ComGC bands, with a band at 20 kDa possibly reflecting dimeric GomGC); right panel shows corresponding immunoblot analysis using EF-Tu-antiserum to ensure equal loading of cell extracts. (TIF) [file pgen.1010696.s005.tif]

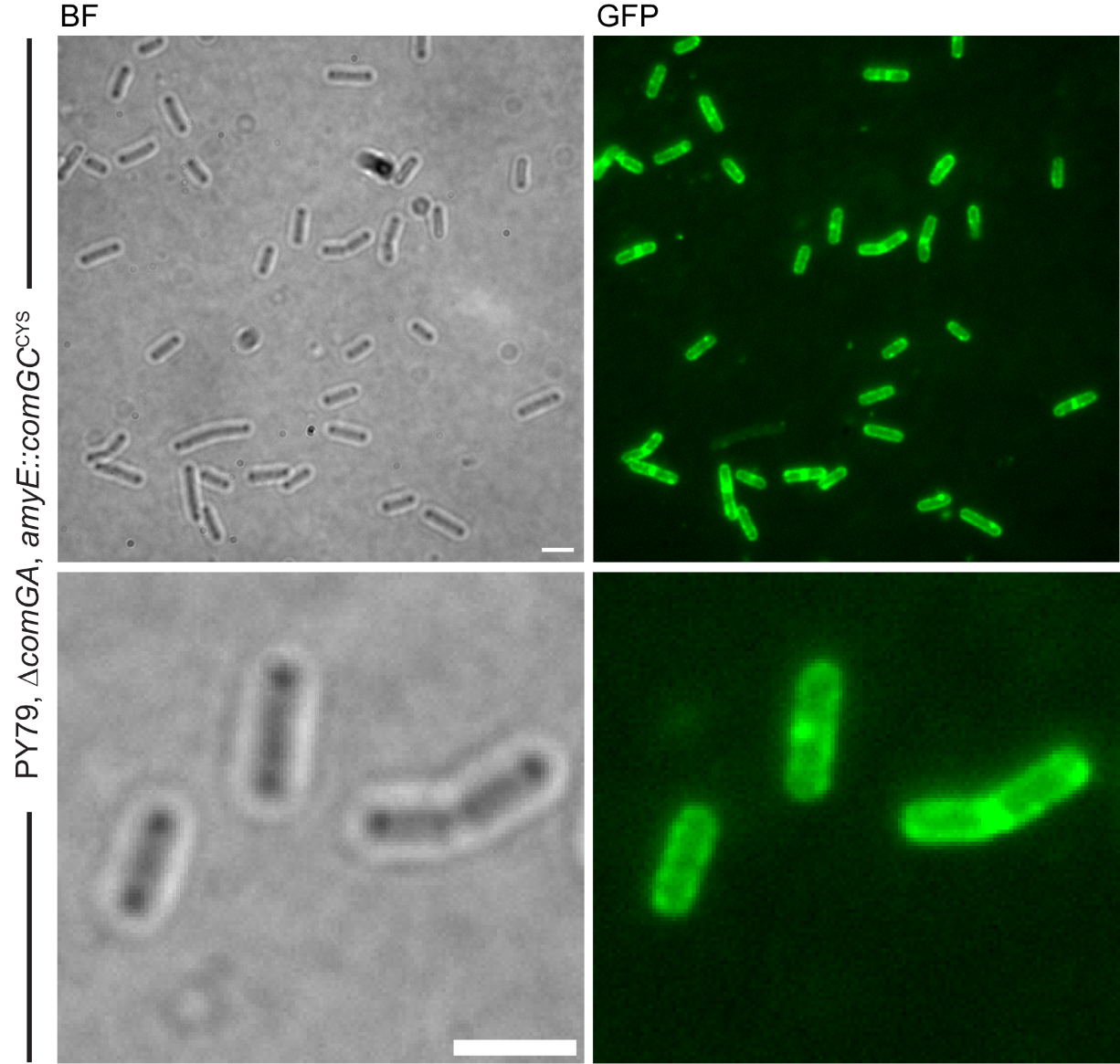

Supplement: S2 Fig — Left images of a panel show bright field images (BF), right panels show epifluorescence pictures (GFP-channel). Scale bars represent 2 μm. (TIF) [file pgen.1010696.s006.tif]

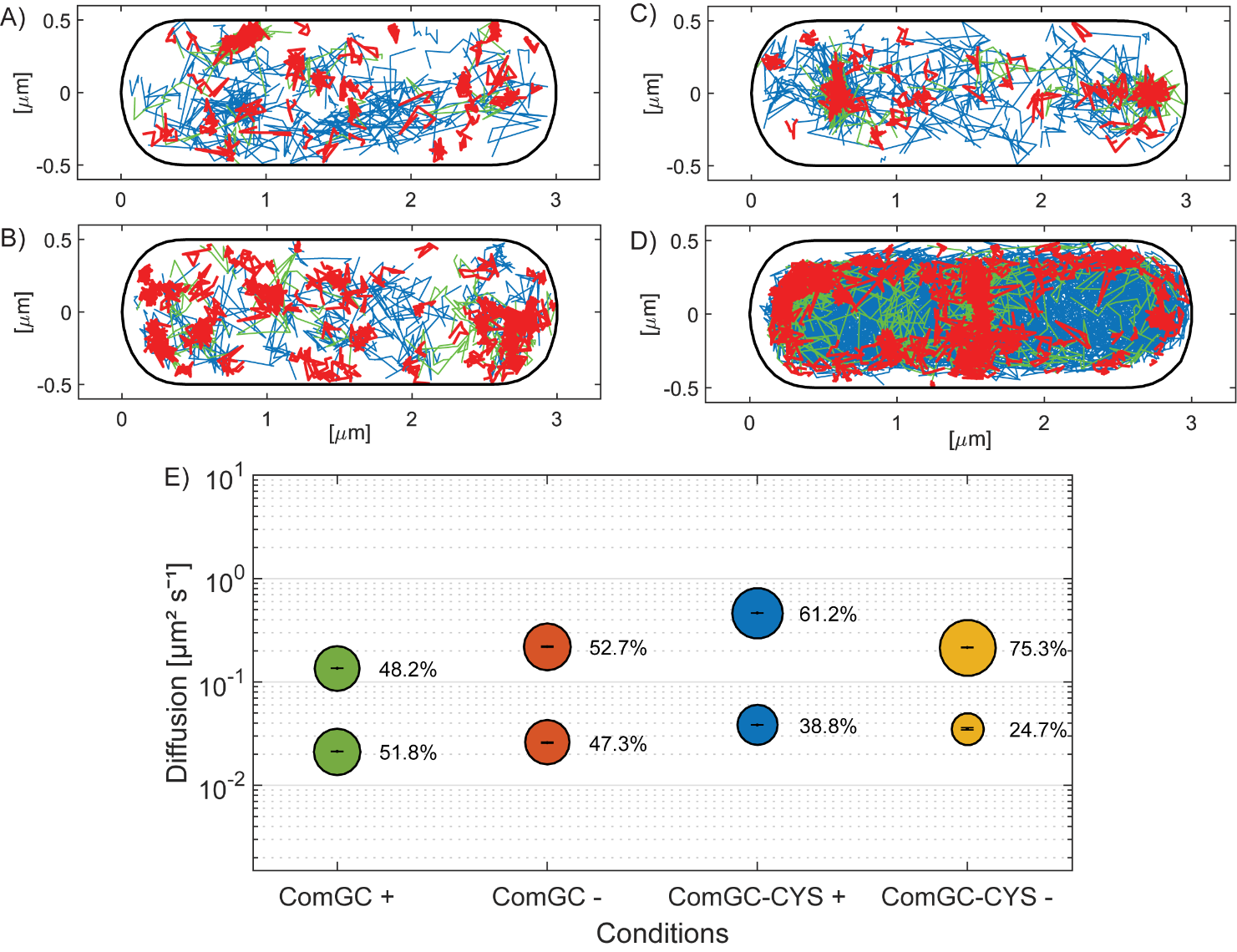

Supplement: S3 Fig — Confinement maps of A) PY79, amyE::comGC with maleimide (A) and without (B), and PY79, amyE::comGCCYS with (D) and without (C) treatment of AF488-C5 maleimide. Confinement radius 108.7 nm (E) Bubble plots show the different sizes for populations of ComGC and ComGC-CYS in cells treated with (+) and without (-) stain. Size of bubbles indicates the size of populations. (TIF) [file pgen.1010696.s007.tif]

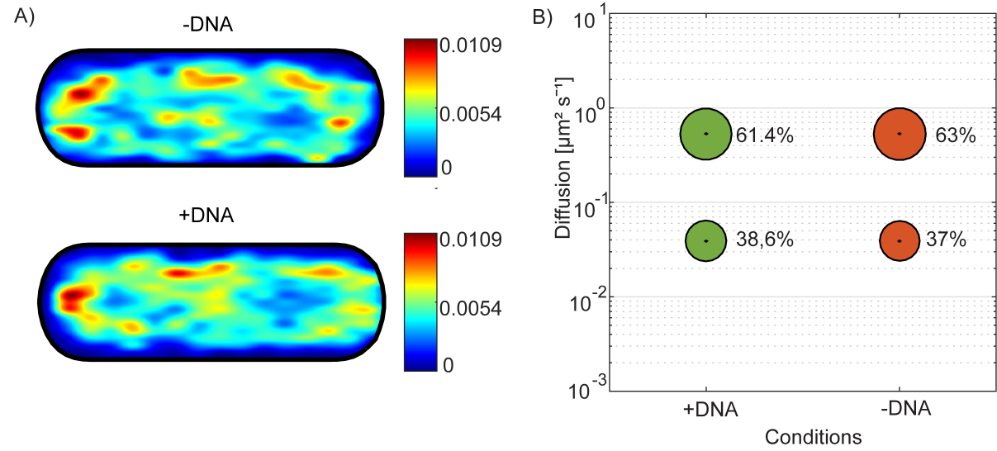

Supplement: S4 Fig — A) Heat maps of ComGC-CYS stained cells with AF488-C5 maleimide with and without incubation of chromosomal DNA (indicated above) in a standardized B. subtilis cell. Confinement radius was 108.7 nm. The colour code on the right indicates the intensity of the signal with presence of the protein. B) Bubble plot of ComGC-CYS with a simultaneous diffusion constant. Cells were treated as indicated (with) DNA for 20 minutes as indicated above. Note that population sizes and diffusion constants are different from Fig 7C, because simultaneous fits were used assuming the same optimal D for both conditions. (TIF) [file pgen.1010696.s008.tif]
