# Supplementary material for: Uptake of environmental DNA in Bacillus subtilis occurs all over the cell surface through a dynamic pilus structure
Source: PLoS Genet. 2023 Oct 10;19(10):e1010696. doi: 10.1371/journal.pgen.1010696 (PMC10564135; doi:10.1371/journal.pgen.1010696)
Supplement: S1 Table — (DOCX) [file pgen.1010696.s009.docx]

Table S1 Oligonucleotides used in this study.

| **Primer** | **sequence (5‘-3‘)** |
| --- | --- |
| ComGC fwd | GAGCTCGCTAGCATGAATGAGAAAGGATTTACACTTG |
| ComGC rev | GAGCTCGCATGCTTAATGTTCAACCTTAACTTCTCCG |
| ComGC RBS fwd | GATTAACTAATAAGGAGGACAAACATGAATGAGAAAGGATTTACACTTG |
| ComGC RBS rev | GCTAGCTGTCGACTAAGCTT |
| ComGC^CYS^ fwd | TGTGAGGGCTATGTGAAAAAGG |
| ComGC^CYS^ rev | CTGTAAATCGGCAAGGCTCG |
| ComGC UP | CTCTGGATCATTGAAGCAGGGAATTCTAGAAGCTTCTGCA |
| ComGC down | GACAGCCGAGCATGGGAGCGCCATGGCATGCATCGATAGA |
| Q5 mut fwd | GCAAGGCTCGGAGTTTGT |
| Q5 mut rev | CGATTTACAGTGTGAGGGCTATG |
