# Supplementary material for: Uptake of environmental DNA in Bacillus subtilis occurs all over the cell surface through a dynamic pilus structure
Source: PLoS Genet. 2023 Oct 10;19(10):e1010696. doi: 10.1371/journal.pgen.1010696 (PMC10564135; doi:10.1371/journal.pgen.1010696)
Supplement: S2 Table — (DOCX) [file pgen.1010696.s010.docx]

Table S2 Plasmids used in this study.

| name | description | resistance | reference |
| --- | --- | --- | --- |
| pDR111 |  | amp | (BGSCID: ECE312) |
| pMAD |  | amp | (1) |
| pAKI1 | pDR111 x *comGC**^CYS^* | amp | this study |
| pAKI2 | pDR111 x *comGC* | amp | this study |
| pAKI3 | pDR111 x *comGC* (RBS) | amp | this study |
| pAKI4 | pMAD x *comGC^CYS^* | amp | this study |

References

1. Ben-Yehuda, S., Rudner, D. Z., & Losick, R. (2003). RacA, a bacterial protein that anchors chromosomes to the cell poles. *Science*, *299*(5606), 532-536
