# Supplementary material for: Uptake of environmental DNA in Bacillus subtilis occurs all over the cell surface through a dynamic pilus structure
Source: PLoS Genet. 2023 Oct 10;19(10):e1010696. doi: 10.1371/journal.pgen.1010696 (PMC10564135; doi:10.1371/journal.pgen.1010696)
Supplement: S3 Table — (DOCX) [file pgen.1010696.s011.docx]

Table S3 *E. coli* and *B. subtilis* strains used in this study

| Strain | genotype | Reference |
| --- | --- | --- |
| PG001 | PY79 | Laboratory strain |
| PG105 | *F´endA1 hsdR17 (rK- mK+) glnV44 thi-1 recA1 gyrA (nalR) relA1 Δ(lacIZYA-argF) U169 deoR (Φ80dlacΔ(lacZ)M15)* | New England Biolabs |
| PG677 | PY79, *comK::kan* | BKK10420 |
| PG3717 | PY79, *comGA::ery* | BKE24730 |
| PG4536 | PY79, *amyE::P_hyperspank_comGC, spec* | This study |
| PG4537 | PY79, *amyE::P_hyperspank_comGC^CYS^, spec* | This study |
| PG4538 | PY79, *comGC^CYS^* | This study |
| PG4539 | DH5α, pDR111 x *comGC^CYS^ (pAKI1)* | This study |
| PG4540 | DH5α, pDR111 x *comGC (pAKI2)* | This study |
| PG4541 | DH5α, pDR111 x *comGC* (RBS) *(pAKI3)* | This study |
| PG4542 | DH5α, pMAD | (1) |
| PG4543 | PY79, *comGC::ery* | BKE24710 |
| PG679 | PY79,∆*comGA* | BD1248 |
| PG4607 | PY79,∆*comGA, amyE::P_hyperspank_comGC^CYS^, spec* | This study |

References

1. Arnaud M., Chastanet A., Debarbouille M. (2004) New vector for efficient allelic replacement in naturally nontransformable, low-GC-content, gram-positive bacteria. *Appl. Environ. Microbiol.* 70:6887-6891. 10.1128/AEM.70.11.6887-6891.2004.
