# Supplementary material for: Uptake of environmental DNA in Bacillus subtilis occurs all over the cell surface through a dynamic pilus structure
Source: PLoS Genet. 2023 Oct 10;19(10):e1010696. doi: 10.1371/journal.pgen.1010696 (PMC10564135; doi:10.1371/journal.pgen.1010696)
Supplement: S4 Table — (DOCX) [file pgen.1010696.s012.docx]

Table S4 Single molecule tracking of ComGC^CYS^ with/ and without addition of DNA with an incubation time of 20 minutes

|  | PY79, *amyE::comGC^CYS^* -DNA | PY79, *amyE::comGC^CYS^+*DNA |
| --- | --- | --- |
| Pop_1_ [%] | 37 | 38.6 |
| Pop_2_ [%] | 63 | 61.4 |
| D_1_ +/- CI [µm^2^/s] | 0.039 ± 0.0 | 0.033 ± 0.001 |
| D_2_ +/- CI [µm^2^/s] | 0.53 ± 0.001 | 0.53 ± 0.001 |
